# Evaluation of antioxidant activity and chemical prospecting of metabolites produced by *Streptomyceshygroscopicus*S75-5

**DOI:** 10.1186/1753-6561-8-S4-P234

**Published:** 2014-10-01

**Authors:** Sandrine Maria de Arruda Lima, Temístocles Ítalo de Santana, Jaciana dos Santos Aguiar, Janete Magali de Araújo, Teresinha Gonçalves da Silva

**Affiliations:** 1 Universidade Federal do Pernambuco, Recife, PE, Brazil

## Background

*Streptomyces hygroscopicus*is a species that produces a variety of antibiotics belonging to different classes. It hás a distinct structure and possesses certain biological properties, such as antimicrobial and antitumor [1]. According to a study by Rong and Huang (2012) [2] more than 650 kinds of bioactive substances have been produced by *Streptomyces hygroscopicus *and species related to it. Members of this clade are still one of the main means of screening new substances. The aim of this study was to investigate the antioxidant activity and chemical profile of metabolites of *Streptomyces hygroscopicus *S75-5 obtained by fermentation in liquid and solid media. The antioxidant activity evaluation was conducted using the DPPH method [3] and chemical prospecting following the methodology described by Harborne [4]. The results showed that the extracts of fermentation in the liquid medium presented the highest percentage of antioxidant activity. Chemical prospecting also made it possible to determine the presence of phenolic compounds in the ethyl acetate extract of the metabolic liquid by fermentation in a liquid medium.

## Methods

*Streptomyces hygroscopicus *S75-5 was grown in an ISP-2 medium liquid for 48 hours (30 °C / 120 rpm) and inoculated into a liquid medium (soybean meal) for 96 hours (30 °C / 120 rpm) and a solid medium (parboiled rice) for 21 days (30°C). After cultured in a liquid medium, the biomass was separated from the liquid metabolic and extracted with methanol. The metabolic liquid was extracted with ethyl acetate. Metanol was also added to the solid medium. The antioxidant activity evaluation was conducted using of the methodology of sequestration free radical 2,2-diphenyl-1-picrylhydrazyl [3], where 80 μL of the extracts and the standard solution, quercetin, were added to 500 μL of a DPPH solution (44 μg/mL). After 30 minutes, the absorbance was read in a UV-Vis spectrophotometer at 517 nm. Each sample was tested in triplicate and the activity expressed in percentages according to the equation: AA% = 100 - {[(Abs extract - Abs blank) × 100] / Abscontrol}. For chemical prospecting of secondary metabolites, thin layer chromatography was used to analyze aliquots of 10 μL of the extracts to detect the presence of alkaloids, reducing sugars, phenolics compounds, flavonoids, tannins, triterpenes and steroids, using revealing specific [4].

## Results and conclusions

In the antioxidant activity, the methanolic biomass extracts, ethyl acetate of the metabolic liquid and methanolic of the solid fermentation showed, respectively, 62.2%; 76.9% and 49.1% capability to sequester the free radical, respectively. The ethyl acetate extract of the metabolic liquid from fermentation in a liquid medium was responsible for the highest percentage of antioxidant activity, making it possible to detect the presence of alkaloids, reducing sugars, phenols, triterpenes and steroids in the chemical prospecting. In general, phenolic compounds, classified as flavonoids and non-flavonoids, are multifunctional, such as antioxidants. These can operate in various ways, for example, by combating free radicals by donation from a hydrogen atom of a hydroxyl group (OH) of the structure aromatic [5].
